# Effects of Kefir on the Cardiac Autonomic Tones and Baroreflex Sensitivity in Spontaneously Hypertensive Rats

**DOI:** 10.3389/fphys.2016.00211

**Published:** 2016-06-07

**Authors:** Brunella F. Klippel, Licia B. Duemke, Marcos A. Leal, Andreia G. F. Friques, Eduardo M. Dantas, Rodolfo F. Dalvi, Agata L. Gava, Thiago M. C. Pereira, Tadeu U. Andrade, Silvana S. Meyrelles, Bianca P. Campagnaro, Elisardo C. Vasquez

**Affiliations:** ^1^Laboratory of Translational Physiology, Federal University of Espirito SantoVitoria, Brazil; ^2^Pharmaceutical Sciences Graduate Program, Vila Velha UniversityVila Velha, Brazil; ^3^Department of Physiology, Federal University of Vale Sao FranciscoPetrolina, Brazil; ^4^Department of Biochemistry, Institute of Education, Science and TechnologyVila Velha, Brazil

**Keywords:** probiotic, hypertension, sympathetic tone, vagal tone, tachycardia, bradycardia

## Abstract

**Aims:** It has been previously shown that the probiotic kefir (a symbiotic matrix containing acid bacteria and yeasts) attenuated the hypertension and the endothelial dysfunction in spontaneously hypertensive rats (SHR). In the present study, the effect of chronic administration of kefir on the cardiac autonomic control of heart rate (HR) and baroreflex sensitivity (BRS) in SHR was evaluated.

**Methods:** SHR were treated with kefir (0.3 mL/100 g body weight) for 60 days and compared with non-treated SHR and with normotensive Wistar-Kyoto rats. Cardiac autonomic vagal (VT) and sympathetic (ST) tones were estimated through the blockade of the cardiac muscarinic receptors (methylatropine) and the blockade of β_1−_adrenoceptor (atenolol). The BRS was evaluated by the tachycardia and bradycardia responses to vasoactive drug-induced decreases and increases in arterial blood pressure (BP), respectively. Additionally, spontaneous BRS was estimated by autoregressive spectral analysis.

**Results:** Kefir-treated SHR exhibited significant attenuation of basal BP, HR, and cardiac hypertrophy compared to non-treated SHR (12, 13, and 21%, respectively). Cardiac VT and ST were significantly altered in the SHR (~40 and ~90 bpm) compared with Wistar rats (~120 and ~30 bpm) and were partially recovered in SHR-kefir (~90 and ~25 bpm). SHR exhibited an impaired bradycardic BRS (~50%) compared with Wistar rats, which was reduced to ~40% in the kefir-treated SHR and abolished by methylatropine in all groups. SHR also exhibited a significant impairment of the tachycardic BRS (~23%) compared with Wistar rats and this difference was reduced to 8% in the SHR-kefir. Under the action of atenolol the residual reflex tachycardia was smaller in SHR than in Wistar rats and kefir attenuated this abnormality. Spectral analysis revealed increased low frequency components of BP (~3.5-fold) and pulse interval (~2-fold) compared with Wistar rats and these differences were reduced by kefir-treatment to ~1.6- and ~1.5-fold, respectively. Spectral analysis also showed an impairment of spontaneous BRS in SHR, but kefir-treatment caused only a tendency to reverse this result.

**Conclusions:** The novelty of this study is that daily chronic consumption of a low dose of kefir reduced the impairment of the cardiac autonomic control of HR and of the impaired BRS in SHR.

## Introduction

In recent years, the fermented milks containing lactic acid bacteria, the so-called probiotics, including kefir, emerged as an alternative therapy due to the growing interest for well-being and healthy lifestyle. Experimental and clinical studies have demonstrated the beneficial effects of functional foods in cardiovascular diseases (Jakala et al., 2009; Turpeinen et al., 2011; Monteiro et al., 2012; Astrup, 2014; Friques et al., 2015).

The spontaneously hypertensive rat (SHR) have been used as an important tool to understanding cardiovascular dysfunctions, such as high blood pressure (BP), endothelial injury and abnormal neural control of the cardiovascular system (Vasquez et al., 1997; Abreu et al., 1998; Blanco et al., 2015; Friques et al., 2015). In recent years, this hypertensive animal emerged as a promising model to identify alternative or non-pharmacological agents for prevention/treatment of cardiovascular disease because it develops essential hypertension (Ceroni et al., 2009; Monteiro et al., 2012; Barbosa Neto et al., 2013; Friques et al., 2015). In this regard, our group has recently demonstrated the beneficial effects of kefir on BP and endothelial dysfunction in chronically treated SHR rats (Friques et al., 2015). The mechanisms of those actions included a partial correction of the reactive oxygen species/nitric oxide imbalance and the partial restoring of the endothelial architecture due to endothelial progenitor cells attraction.

Another relevant characteristic of the SHR is an abnormal autonomic nervous control of cardiac activity (Ceroni et al., 2009; Monteiro et al., 2012; Barbosa Neto et al., 2013), which is closely correlated with end-organ damage (Su and Miao, 2005; Abboud, 2010; Abboud et al., 2012). The influence of the autonomic nervous system on the heart rate (HR) and blood pressure (BP) in SHR has clearly been demonstrated (Schenberg et al., 1995; Vasquez et al., 1997; Abreu et al., 1998; Dias da Silva et al., 2002; Silva et al., 2009; Abboud, 2010) and appears to be frequency-dependent (Dias da Silva et al., 2002; Ceroni et al., 2009; Silva et al., 2009). Despite some discrepancies, multiple studies have demonstrated a decreased cardiac parasympathetic (vagal) tone and an increased cardiac and vessel sympathetic tone in SHR when compared with normotensive Wistar rats (Ceroni et al., 2009; Silva et al., 2009; Hayward et al., 2012; Barbosa Neto et al., 2013). However, a possible beneficial action of the probiotic kefir on the cardiac dysautonomia in hypertensive rats has not yet been evaluated.

The baroreflex sensitivity, which is a marker of the capability of reflexes to increase vagal activity and to decrease sympathetic activity in response to a sudden increase in BP, is diminished in experimental models of essential (Barbosa Neto et al., 2013) and secondary (Moyses et al., 1994; Campagnaro et al., 2012) hypertension, as well as in cardiovascular related diseases, such as atherosclerosis (Vasquez et al., 2012). This reflex has classically been evaluated through invasive approaches using vasoconstrictor and vasodilator agents (Moyses et al., 1994; Campagnaro et al., 2012) and later through procedures using spectral analysis (Dias da Silva et al., 2002; Chapleau and Sabharwal, 2011).

The concept that the BP is affected by baroreflex-mediated changes in autonomic nerve activity in the heart and systemic vasculature, highlights the importance of studying the effects of alternative functional foods therapies on the autonomic and reflex control of the cardiovascular system. Therefore, the present study was designed to test the hypothesis that chronic administration of kefir can ameliorate the abnormal autonomic control of HR and the impaired baroreflex sensitivity in SHR rats.

## Material and methods

### Animals

The present study was performed in male 4-month-old SHR and in age-matched Wistar-Kyoto rats obtained from the Vila Velha University animal care and that were maintained in the animal care facility of the Federal University of Espirito Santo, Brazil. The rats were housed in individual acclimatized plastic cages with a controlled temperature (22–23°C), light-dark cycle (12:12-h), and were fed with a standard rat chow and water *ad libitum.* The study protocols were previously approved by the Institutional Committee on Animal Care (CEUA-UFES, Protocol #040/2014). All experimental procedures were performed in accordance with the guidelines for the care and use of laboratory animals as recommended by the National Institutes of Health (NIH).

### Kefir preparation, identification, and administration

The identification, preparation and administration of kefir were performed as previously described (Friques et al., 2015). Briefly, kefir was obtained from the fermentation of the grains in whole milk. The kefir beverage was prepared by adding kefir grain to pasteurized whole milk in a ratio of 4% (w/v).

The treatment of the animals was started at the age of 4-month-old and lasted 60 days. One group of animals was treated with kefir (0.3 mL/100 g body weight, by gavage, SHR-kefir) for 60 days. Another group of SHR was administered whole milk (0.3 mL, pH adjusted to 4.5, SHR) for 60 days for use as the hypertensive controls. The rationale for using 0.3 mL/100 g body weight was based on the dose translation from human to animal studies by a simple method using the body surface area normalization and that dose is compatible with that used in human beings. The Wistar rats were administered whole milk for 60 days and were used as normotensive control groups. The reason for treating the animals with kefir for 60 days was based on a previous study from our group (Friques et al., 2015), demonstrating that the treatment for less than 60 days had no effect on cardiovascular parameters in SHR.

### Instrumentation for hemodynamic measurements

After 60 days of kefir administration, the animals were intraperitoneally anesthetized with a mixture of ketamine and xylazine (91+9.1 mg/kg) and a polyethylene catheter (PE 50) was positioned into the femoral vein for injection of drugs and another into the inferior aorta for measurement of pulsatile pressure, mean BP and HR 48 h later using a data-acquisition system (Biopac Systems, Santa Barbara, CA, USA) in unrestrained animals.

At the end of the evaluation of hemodynamic parameters, the animals were euthanized by an over-dose of thiopental (100 mg/kg) and the hearts were excised. The right and the left ventricles, including the interventricular septum, were dissected from the remaining cardiac tissues. The tibia bone was also dissected and its length was measured. To evaluate the extent of cardiac hypertrophy for each animal the left ventricle weight was normalized by the animal's body weight and tibia length.

### Evaluation of the cardiac autonomic tones

Cardiac autonomic tones were estimated through selective pharmacological blockers of the muscarinic receptors (methylatropine) and the β_1−_adrenergic receptors (atenolol) in conscious animals, based on previous studies from our laboratory and from others (Chapleau and Sabharwal, 2011; Campagnaro et al., 2012). As shown in the scheme in **Figure 3** (top), the cardiac parasympathetic tone was estimated by the change in basal HR 15 min after a single injection of methylatropine (1 mg/kg, i.v.), which reaches a plateau effect at approximately this time and lasts for ~30 min. Immediately afterwards, they were injected with atenolol (1 mg/kg, i.v.), which also reached a maximum effect 15 min later, and this value was considered the intrinsic (pacemaker) HR. the next day, the sequence of the injections was the opposite, and the cardiac sympathetic tone was estimated by the change in the basal HR 15 min after atenolol, and the intrinsic HR was estimated 15 min under the double blockade.

### Pharmacological baroreflex sensitivity

The baroreflex control of arterial pressure was evaluated in conscious animals by measuring the tachycardia and bradycardia in response to an equivalent increase in arterial BP in each of the three groups of conscious animals. Based on previous studies from our laboratory demonstrating that the major sensitivity of the baroreflex is at changes in arterial BP close to the resting values and that changes in BP higher than 40 mmHg could elicit complex humoral mechanisms, we decided to challenge the baroreflex with sudden increase and decrease in BP of ~25 mmHg (Schenberg et al., 1995; Gava et al., 2004). These changes in BP were elicited by a single bolus injection of phenylephrine (1 μg/kg) and sodium nitroprusside (1 μg/kg), respectively.

The relative contribution of the cardiac parasympathetic and sympathetic nerves was assessed by increase or decrease in HR in response to a sudden decrease or increase in BP, under the blockade of cardiac muscarinic receptors with methylatropine (1 mg/kg, i.v.) on day one and the blockade of the cardiac β_1_-adrenoceptors with atenolol (1 mg/kg, i.v.) on the following day.

### Spectral analysis

An aim of this protocol was to characterize patterns of autonomic control in SHR and normotensive Wistar rats by power spectral analysis of pulse interval (PI) variability and BP variability as previously described (Dantas et al., 2012), Power spectra analysis was performed using a Matlab-customized software validated against the software developed by A. Porta in Italy (Linear Analysis version 8.3, University of Milan, Italy). Pulse intervals from systolic arterial pressure were obtained from 30 min of continuous BP records in conscious animals. A parametric method based on autoregressive model of spectral estimation was performed for systolic arterial BP and PI analysis. The oscillatory components in the rat were quantified as low frequency (LF: 0.2–0.8 Hz) mainly corresponding to sympathetic activity and high frequency (HF: 0.8–2.8 Hz) corresponding to vagal activity (influenced by respiration; Silva et al., 2009; Dantas et al., 2013; Quagliotto et al., 2015).

The estimation of spontaneous baroreflex sensitivity was obtained by measuring oscillations in BP and PI in the LF range using spectral analysis. The baroreceptor sensitivity value was provided by α-LF index, which was calculated as the square root of the ratio between the absolute power of PI-LF/BP-LF, expressed in ms/mmHg. The oscillations in BP and PI needed to be coherent (coherence^2^), with a coherence higher than 0.5, and a negative phase difference was required between the two variables, as recently reviewed (Chapleau and Sabharwal, 2011).

### Statistical analysis

The values are expressed as means ±SEM. First, a D'Agostino-Pearson omnibus normality test was performed to verify if that the values came from a Gaussian distribution. All data Statistical comparisons between the different groups were performed by a randomized one-way analysis of variance (ANOVA), followed by Bonferroni's *post hoc* test. A value of *p* < 0.05 was considered statistically significant. Statistical analysis was performed using GraphPad Prism software version 6.07 (GraphPad, Inc., San Diego, CA, USA).

## Results

### Microbiological analysis of kefir

The microbiological analysis of random samples of grains used in the present study, showed a dominant microflora of kefir, which included various bacteria that are known to have beneficial effect (*Acetobacter aceti, Acetobacter sp., Lactobacillus delbrueckii delbrueckii, Lactobacillus fermentum, Lactobacillus fructivorans, Enterococcus faecium, Leuconostoc spp.*), as well as *Lactobacillus kefiranofaciens*, and yeasts (*Candida famata, Candida krusei*, and *Candida kefir*). The global counting of microorganisms in the samples of milk fermented with kefir grains was 7.6 x 10^7^ units forming colonies (UFC)/mL. Fermenter microorganisms averaged 6.6 × 10^7^ UFC/mL. *Lactobacillus sp* and *Lactococcus sp* represent the largest and most commonly identified lactic acid bacteria isolates. The average number of acetic and fermenter bacterias was 2.0 × 10^6^ and 6.6 × 10^7^ UFC/mL, respectively and the average number of yeasts was ~4.1 × 10^5^ UFC/mL.

### Basal BP and HR in conscious animals

**Figure 1:**
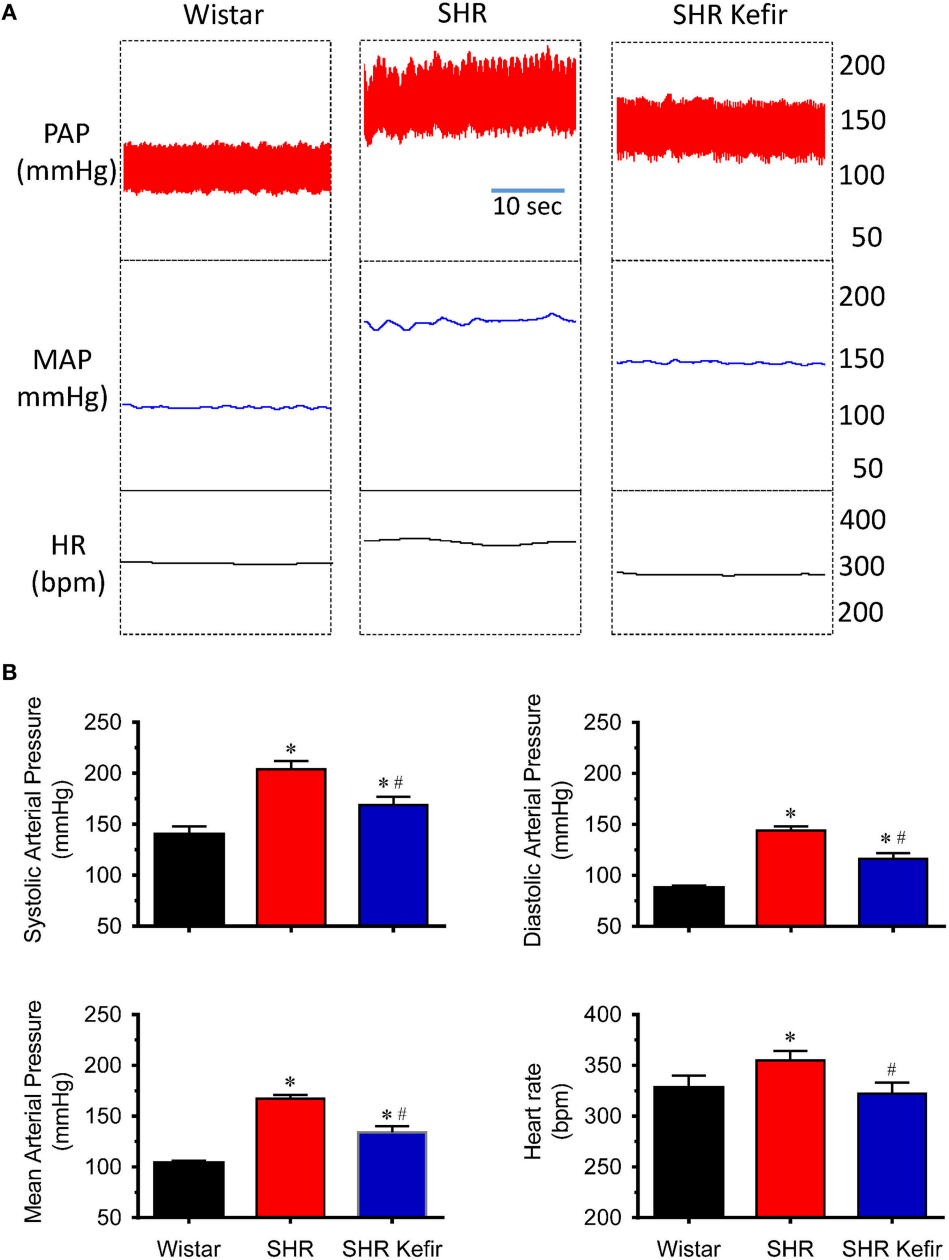
**Effects of chronic administration of kefir on the pulsatile (PAP) and mean (MAP) arterial pressure and heart rate (HR) in conscious spontaneously hypertensive rats (SHR)**. Top panels **(A)** show representative recordings of PAP, MAP, and HR. Bar graphs **(B)** values are means ± SEM (*n* = 12 per group). ^*^p < 0.5 compared to Wistar group, ^#^*p* < 0.05 compared to non-treated SHR (one-way ANOVA).

As expected, the conscious SHR exhibited significant high levels of systolic, diastolic and mean BP (204 ± 8, 144 ± 4, and 167 ± 4 mmHg, respectively, *p* < 0.05) and resting HR (355 ± 9 bpm, *p* < 0.05) compared with the Wistar rats (140 ± 8, 88 ± 2, and 104 ± 2 mmHg, respectively, and 328 ± 12 bpm; Figure 1). Administration of kefir for 60 days caused a significant attenuation of systolic, diastolic and mean BP which was reduced to 169 ± 8, 116 ± 6, and 134 ± 6 mmHg, respectively, and on HR, which was reduced to 322 ± 11 bpm.

### Body weight and cardiac hypertrophy

**Figure 2:**
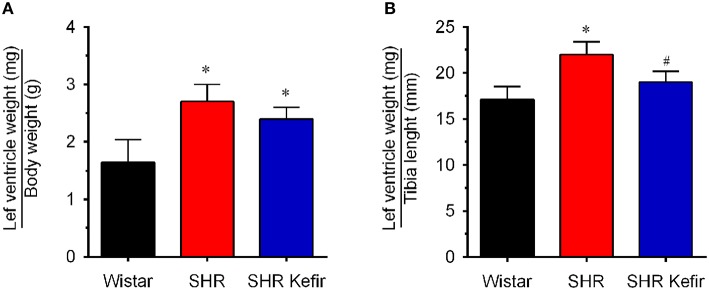
**Effects of chronic administration of kefir on the cardiac hypertrophy in SHR based on the left ventricular weight normalized by the body weight (A: left graph) and tibia length (B: right graph)**. Values are means ± SEM (*n* = 8 per group). ^*^p < 0.05 compared to Wistar group, ^#^*p* < 0.05 compared to non-treated SHR (one-way ANOVA).

Administration of kefir did not cause effect on the consumption of water and food. At the moment of the evaluation of the outcomes (i.e., 60 days after kefir administration), body weight was significantly diminished (~32%, *p* < 0.05) in non-treated and kefir-treated SHR than in the age-matched Wistar rats (328 ± 3 vs. 480 ± 8 g, *p* < 0.05). Left and right ventricular cardiac mass were significantly (*p* < 0.05) greater in non-treated SHR (893 ± 30 mg or 13% and 386 ± 24 mg or 40%, respectively) than in normotensive Wistar rats (787 ± 29 and 274 ± 20 mg, respectively). Kefir administration for 60 days caused a significant and complete normalization in the left ventricle mass (785 ± 25 mg or –13%) and a significant attenuation of the right ventricle mass (352 ± 37 mg or −10%). The normalization of the left ventricle weight by the body weight and tibia length revealed left ventricular hypertrophy in the non-treated SHR group (2.7 ± 0.3 mg/g and 22 ± 1.4 mg/mm, respectively, *p* < 0.05) compared with the values observed in the Wistar group (1.6 ± 0.4 mg/g and 17 ± 1.4 mg/mm, respectively; Figure 2). The treatment of SHR with kefir for 60 days had no significant effect on the left ventricle hypertrophy when the ventricle chamber weight was normalized by the body weight (2.4 ± 0.2 mg/g and 19 ± 1.2 mg/mm, respectively, *p* < 0.05). However, kefir administration reduced the left ventricle hypertrophy, when the ventricle chamber weight was normalized by the tibia length, which was 41 ± 0.3 mm in both SHR and SHR-kefir vs. 46 ± 0.3 mm in Wistar rats, *p* < 0.05).

### Cardiac autonomic tone

**Figure 3:**
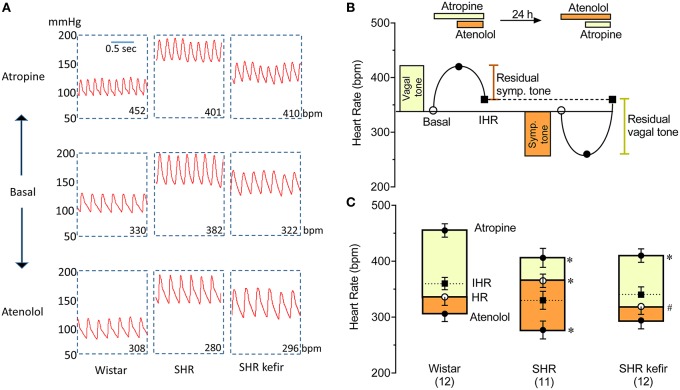
**Effects of chronic administration of kefir on the cardiac autonomic tones in SHR**. **(A)**: typical recordings of pulsatile blood pressure before and respective chronotropic changes after the blockade of muscarinic receptors with methylatropine (1^st^ day) and after the blockade of β-adrenoceptors (2^nd^ day). **(B)**: scheme showing the estimation of the vagal tone through methylatropine and the sympathetic tone through atenolol and the pacemaker or intrinsic heart rate (IHR), which was determined by the double blockade of vagal and sympathetic activity. **(C)**: mean values ± SEM (*n* = 11 to 12 per group) used for determination of each parameter. ^*^p < 0.05 vs. Wistar group; ^#^*p* < 0.05 vs. non-treated SHR. **(B)**: upper panel was redrawn from Chapleau and Sabharwal (2011).

The protocol used to assess the cardiac parasympathetic (vagal) and sympathetic tones is schematically shown in Figure 3B. Figure 3A shows representative recordings of arterial BP and HR and Table 1 and Figure 3C show the average data. In the normotensive Wistar group, the blockade of muscarinic receptors with methylatropine resulted in a significant increase in basal HR, from 336 ± 15 to 455 ± 11 bpm, indicating a vagal tone of +120 bpm (Figure 3C). In the non-treated SHR group, the change in basal HR was from 365 ± 12 to 406 ± 17 bpm (approx. +40 bpm), i.e., a significant decreased vagal tone compared with Wistar rats (Table 1 and Figure 3C). In the SHR group treated with kefir for 60 days, methylatropine caused a significant change from 319 ± 15 to 410 ± 12 bpm (Table 1 and Figure 3C), indicating a partial recovery of the vagal tone (approximately +90 bmp, *p* < 0.05; Figure 3C).

**Table 1:** **Effects of methylatropine and atenolol on resting heart rate in conscious Wistar, non-treated SHR and SHR treated for 60 days with kefir**.

**Heart Rate (bpm) Measurement**	**Groups**		
	**Wistar (12)**	**SHR (12)**	**SHR kefir (11)**
Basal	336 ± 15	365 ± 12^*^	319 ± 15^#^
Methylatropine effect (muscarinic blockade)	455 ± 11	406 ± 17^*^	410 ± 12^*^
Atenolol effect (β-adrenoceptor blockade)	306 ± 14	277 ± 16^*^	294 ± 15
Double blockade effect	360 ± 11	330 ± 16	340 ± 14

*Values are means ± SEM*.

* p < 0.05 vs. Wistar group;

# *p < 0.05 vs. SHR group*.

The effects of the blockade of β_1_-adrenoceptors with atenolol, which was used to evaluate the cardiac sympathetic tone, is illustrated in Figure 3A and the average data are shown in Table 1 and Figure 3C. The basal HR was reduced to 306 ± 14 bpm (approximately −30 bpm) in the Wistar group. In the non-treated SHR the magnitude of reduction in basal HR was significantly higher (277 ± 16 bpm, approximately −90 bpm, *p* < 0.05), but the treatment of SHR with kefir for 60 days abolished this difference between strains; the basal HR was reduced to 294 ± 15 bpm (approximately −25 bpm).

The intrinsic HR, which was considered as the resting HR under the double blockade with methylatropine and atenolol (Table 1), was similar in the three groups of animals (Figure 3C).

### Classical pharmacological analysis of the baroreflex function

**Figure 4:**
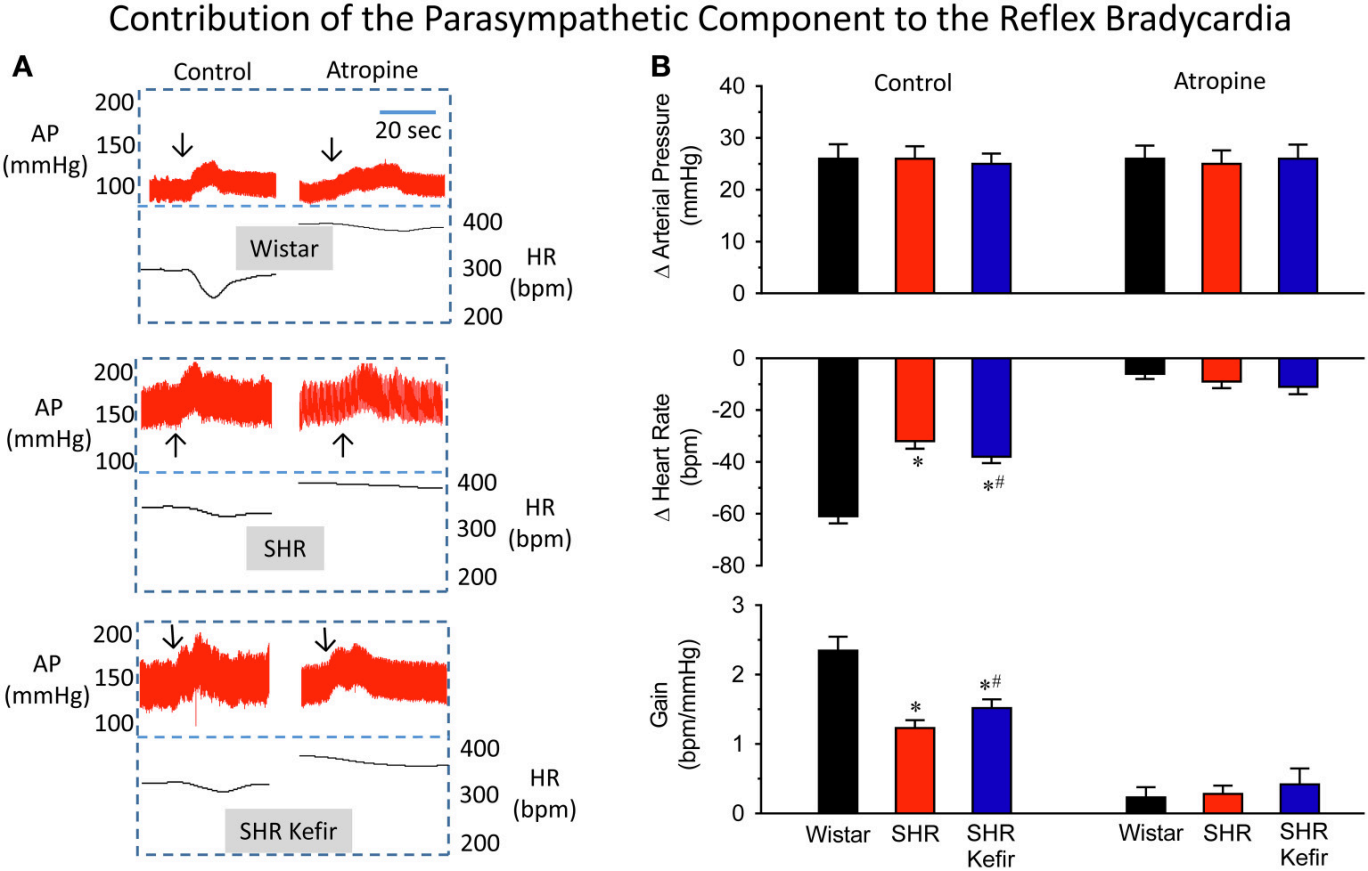
**Effects of chronic kefir administration on the reflex bradycardia in SHR compared with non-treated SHR and normotensive Wistar rats**. In the three groups of conscious animals, similar phenylephrine-induced increases (~25 mmHg) in arterial blood pressure (AP) were used **(A)**: representative recordings of AP and the calculated heart rate (HR) before and after the blockade of the muscarinic receptors with methylatropine. **(B)**: bar graphs sowing means values ± SEM (*n* = 10 to 12 per group) of changes in AP, HR and baroreflex gain. Arrows indicate the moment that the vasoconstrictor was injected. ^*^p < 0.05 vs. Wistar group; ^#^*p* < 0.05 vs. non-treated SHR.

**Figure 5:**
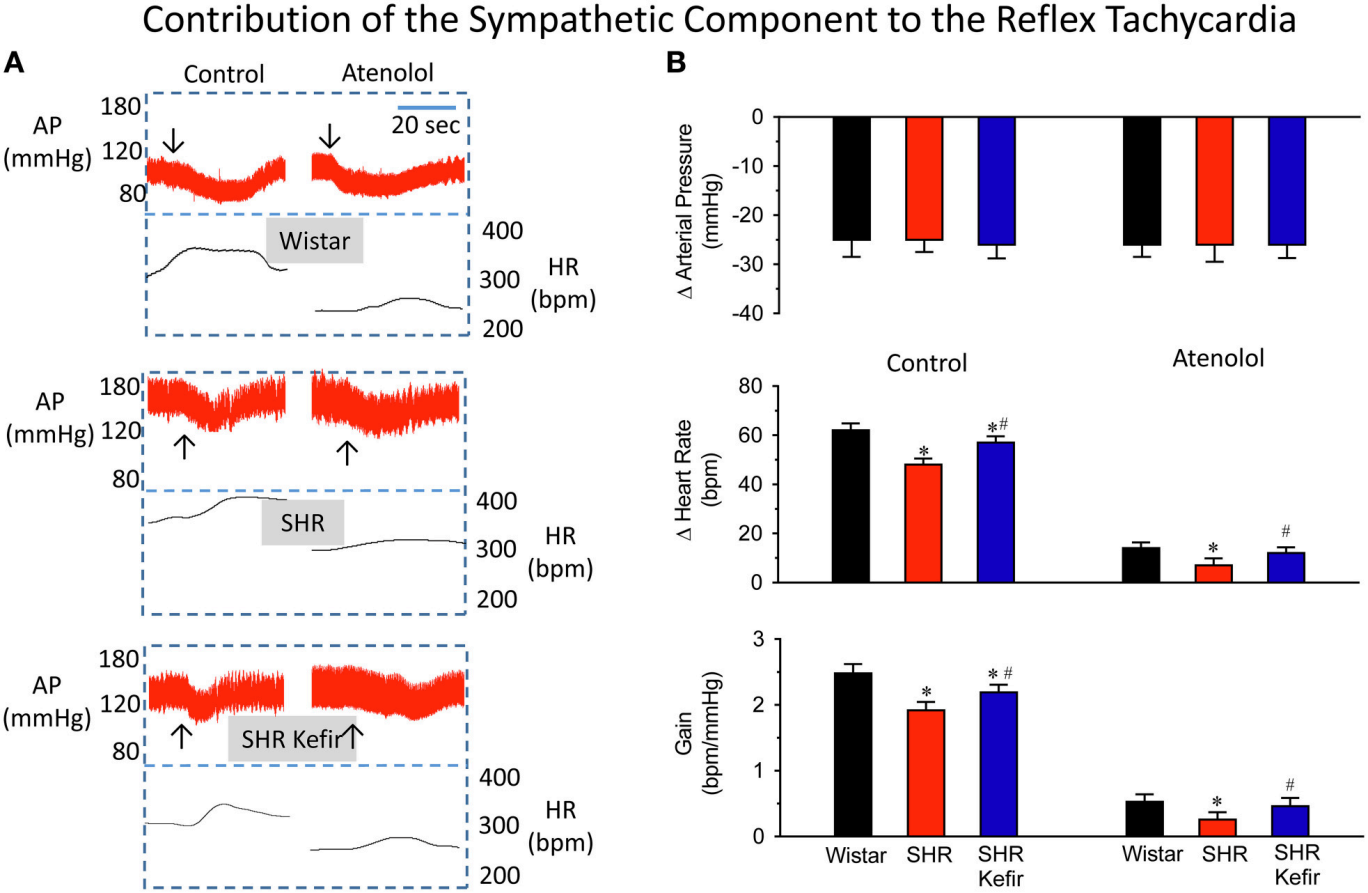
**Effects of chronic kefir administration on the reflex tachycardia in SHR compared with non-treated SHR and normotensive Wistar rats**. In the three groups of conscious animals, similar sodium-nitroprusside-induced increases (~25 mmHg) in arterial pressure (AP) were used **(A)**: representative recordings of AP and the calculated heart rate (HR) before and after the blockade of the β_1_-adrenoceptors with atenolol. **(B)**: bar graphs sowing means values ± SEM (*n* = 10 to 12 per group) of changes in AP, HR and baroreflex gain. Arrows indicate the moment that the vasodilator was injected. ^*^p < 0.05 vs. Wistar group; ^#^*p* < 0.05 vs. non-treated SHR.

The effect of kefir adminsitration on the baroreflex sensitivity as well as the relative contribution of the cardiac vagal and sympathetic components in conscious SHR are shown in Figures 4, 5. As expected and illustrated in Figure 4, when the baroreflex was challenged by moderate phenylephrine-induced increases in arterial BP (approx. 25 mmHg), the SHR group showed a significantly decrese in the reflex bradycadia (~50%, *p* < 0.05), when compared with the Wistar group and, consequently, a significant reduction in the baroreflex gain (~50%, *p* < 0.05) was observed (Figure 4B). When SHRs were treated with kefir for 60 days, phenylephrine-induced matched-increases in BP resulted in mild, but significant, improvement of the reflex bradycardia (~40%, *p* < 0.05) and consequently also the baroreflex gain (~35%, *p* < 0.05), respectively. The blockade of cardiac muscarinic receptors with methylatropine basically abolished the reflex bradycardia and the baroreflex gain in a similar way in the three groups. The disappearence of intergroup differences in the reflex bradycardia after methylatropine indicate that they were due to the vagal component of the baroreflex.

Figures 5A,B show representative recordings and average data of the baroreflex when it was challenged by a moderate dose of sodium nitroprusside. This vasodilator induced falls in arterial BP of approximatly 25 mmHg in all groups of animals. The SHR group showed a significantly lower reflex tachycadia (23%, *p* < 0.05), when compared with the Wistar group and, consequently, a significant reduction in the baroreflex gain (23%, *p* < 0.05) was observed (Figure 5B). When SHRs were treated with kefir for 60 days, these differences were reduced to ~8% and ~12%, despite the same size of nitroprusside-induced reductions of BP. The blockade of cardiac β_1_-adrenoceptors with atenolol reduced (but not abolished) the reflex tachycardia in Wistar, SHR and SHR-kefir. The values reached were significantly lower in SHR and significantly recovered in the SHR treated with kefir for 60 days (14 ± 2.4, 7 ± 2.8, and 12 ± 2.4 bpm, respectively). A similar effect was observed in the baroreflex gain (0.53 ± 0.11, 0.26 ± 0.10, and 0.46 ± 0.12 bpm/mmHg, respectively; Figure 5). The lower reflex tachycardia and baroreflex gain in SHR under the atenolol effect indicate that part of the reflex tachycardia may be due to a withdrawall of the vagal activity, which was reduced in the SHR.

### Spectral analysis

**Figure 6:**
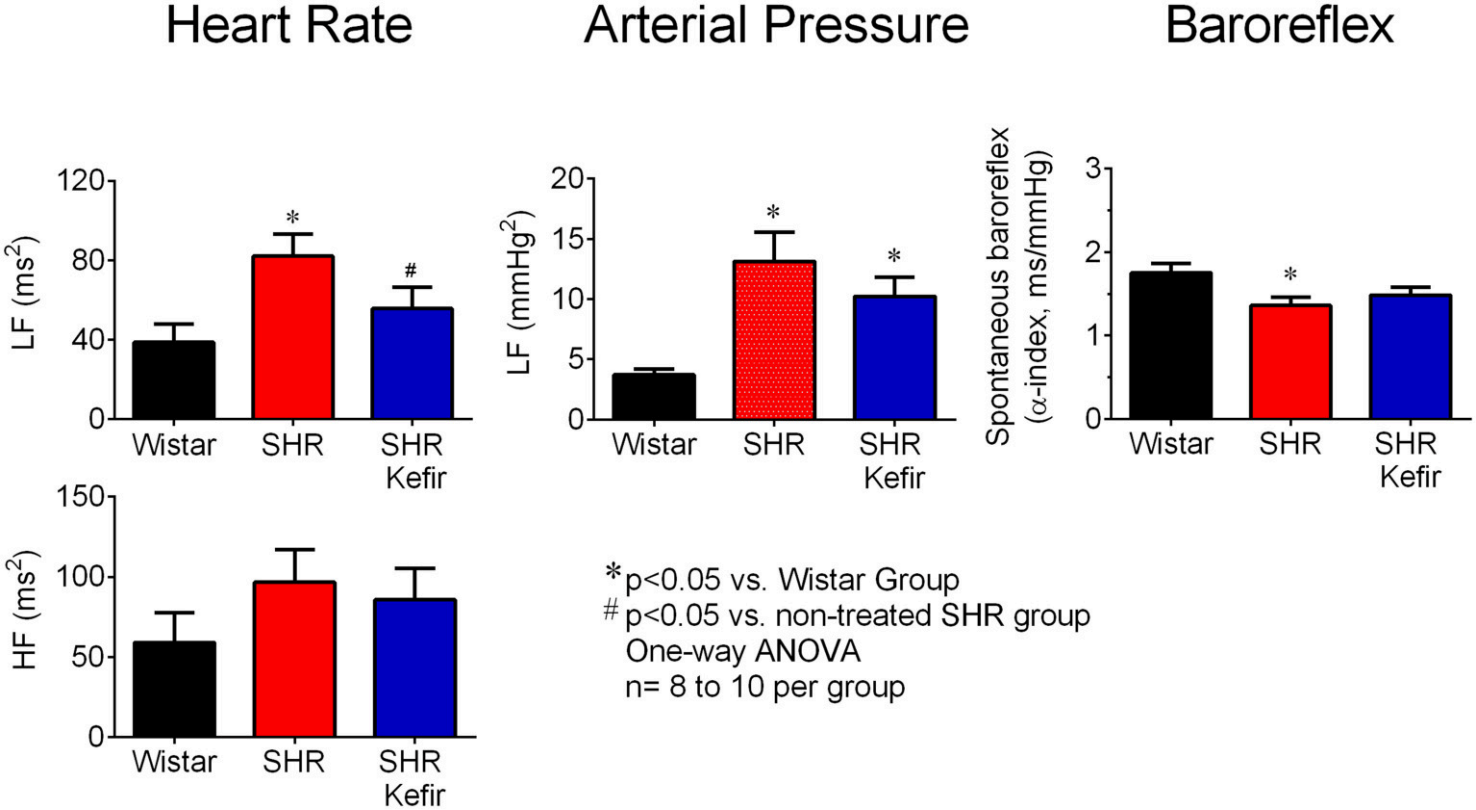
**Effects of chronic kefir administration on the spectral analysis of heart rate and blood pressure in SHR compared with non-treated SHR and normotensive Wistar rats**. Values are means ± SEM (*n* = 10 to 12 per group). ^*^p < 0.05 vs. Wistar group; ^#^*p* < 0.05 vs. non-treated SHR.

Spectral analysis was applied to study the effect of kefir on the PI and BP variability in SHR-kefir compared to non-treated SHR and normotensive Wistar rats. The data showed a significantly higher PI variance in non-treated SHR (730 ± 150 ms^2^, *p* < 0.05) when compared with SHR-kefir (478 ± 80 ms^2^) and with normotensive Wistar rats (247 ± 46 ms^2^). The power spectral analysis of each component of the PI variabilities showed a remarkable difference in that the PI-LF component, which is widely accepted as an index of sympathetic modulation. SHR showed a significant increase (2-folf, *p* < 0.01) in PI-LF when compared with the Wistar group (39 ± 9 ms^2^), suggesting a high sympathetic activity in the hypertensive group. The treatment of SHR with kefir for 60 days caused a significant attenuation of the PI-LF component (~1.4-fold, *p* < 0.05; Figure 6, left bar graph). However, when comparison was made using the normalized values of PI-LF, no significant differences were observed among the three groups of animals, suggesting that this result is consistent with there being no difference between SHR and WKY in sympathetic control of the heart. The comparison of values of PI-HF, which is widely associated with vagal modulation, did not show significant differences comparing the row data (Figure 6, right bar graph).

The spectral analysis of the BP variability showed a significantly higher variance in the non-treated SHR (73 ± 9 mmHg^2^, *p* < 0.05) when compared with Wistar rats (22 ± 3 mmHg^2^) and it was reduced, but not normalized, in the SHR-kefir group (65 ± 8 mmHg^2^). The BP-LF value, which is linked to vasosympathetic tone, was greater in the non-treated SHR group (13 ± 2 mmHg^2^, *p* < 0.05) when compared with the normotensive Wistar rats (3.7 ± 0.4 mmHg^2^; Figure 6, right bar graph), providing an evidence of a relationship between the high BP, high vascular sympathetic activity and power spectra BP-LF in the SHR. Kefir treatment of SHR for 60 days significantly reduced that value to 8 ± 2 mmHg^2^ (Figure 6, right bar graph).

The analysis of the spontaneous baroreflex sensitivity through the LF α-index in the conditions of a coherence higher than 0.5 and a negative phase, showed that the non-treated SHR exhibited significantly lower sensitivity values than the Wistar group (1.36 ± 0.09 vs. 1.75 ± 0.11 ms/mmHg, *p* < 0.05). Kefir treatment of SHR for 60 days resulted in a tendency (1.48 ± 0.09 ms/mmHg, *p* > 0.05) to attenuation of that dysfunction (Figure 6).

## Discussion

The present study investigated the effects of multi-strain kefir on cardiovascular autonomic control and baroreflex sensitivity in SHR rats, since most of the available studies have focused on isolated bacteria providing limited information on the functional role of the widely consumed beverage obtained by fermentation of milk with kefir grains.

As previously demonstrated by us through microbiological analysis and scanning electron microscopy, the Brazilian kefir grains used in the present study are a complex and a sharing symbiotic mixture of beneficial bacteria and yeasts, including the *Lactobacillus kefiranofaciens, Lactobacillus kefir*, and *Candida kefir* (Friques et al., 2015). It has been demonstrated that the chronic consumption of kefir has protective effect against the development of high BP, endothelial dysfunction and endothelial surface damage in the SHR model of essential hypertension (Friques et al., 2015).

Despite a large body of evidence supporting the anti-hypertensive effects of kefir, it should be take into account that these effects may vary depending on the bacterial strain (Million et al., 2012). Most studies have been conducted using different isolated bacterial strains. For instance, it has been demonstrated that the antihypertensive effects of probiotics Lactobacillus strains in SHR could be through a decrease in pro-oxidative status (Gómez-Guzmán et al., 2015). One of the pro-oxidative contributors could be the activation of the renin-angiotensin system, but in the present study, we did not evaluate the effects of kefir on the angiotensin II in the SHR model. However, the widely consumed kefir beverage presents different microorganisms genera and, in this regard, the probiotic treatment protocol applied in the present work are more like the way humans use kefir.

There is emerging evidence of beneficial effects of probiotics in cardiovascular system. However, it is unclear if the promising positive effects evidenced could be due the regulation of cardiovascular autonomic control. We hypothesized that chronic kefir consumption for 60 days may be beneficial in management of autonomic control, based on our previous study showing that administration for shorter periods did not have significant effects on HR, BP and endothelial function (Friques et al., 2015). Similarly, the present study showed that the chronic administration of kefir for 60 days attenuated high BP and consequently the cardiac hypertrophy. Although the reduction in cardiac hypertrophy could be due to a reduction in afterload and/or in cardiac sympathetic tone, additional studies are necessary to elucidate the mechanism by which kefir administration attenuates the cardiac hypertrophy in this model of hypertension.

Considering that, the imbalance of autonomic nervous system markedly influences the HR and BP and that it has been associated with targeted-organ damage and increased risk of morbi-mortality (Abboud, 2010; Abboud et al., 2012), we assessed the effects of kefir on the cardiac parasympathetic (vagal) tone and cardiac and vascular sympathetic tones in SHR by using two different experimental approaches. First, the parasympathetic cardiovagal and the cardio-vaso-sympathetic tones were evaluated through the classic invasive method illustrated in Figure 3. The increase in HR after administering methylatropine, a muscarinic cholinergic receptor blocker, reflects the cardiovagal tone present under baseline resting conditions, and the decrease in HR after atenolol administration, a cardiac β_1_-blocker, reflects cardiac sympathetic tone; a double blockade enables the determination of the intrinsic HR. The imbalance between the cardiac vagal activity (decreased) and sympathetic activity (augmented) in SHR is in agreement with other studies (Barbosa Neto et al., 2013). The novelty of the present study is the finding that chronic administration of kefir caused a significant attenuation of the cardiac autonomic imbalance of a similar magnitude to that obtained by physical exercise (Barbosa Neto et al., 2013), phototherapy (Monteiro et al., 2012), and pharmacological medication (Dias da Silva et al., 2002).

It has been shown that a disruption in the balance between vagal and sympathetic tones can lead to an impairment in baroreflex sensitivity, as has been demonstrated in different models of arterial hypertension (Moyses et al., 1994; Campagnaro et al., 2012), including the SHR (Dias da Silva et al., 2002; Monteiro et al., 2012). Therefore, the effect of kefir on the impaired baroreflex that characterizes SHR was tested in a separated set of experiments using the classical invasive pharmacological maneuvers by which reflex bradycardia during phenylephrine-induced increases in BP and reflex tachycardia during sodium nitroprusside-induced decreases an in BP are analyzed (Moyses et al., 1994; Campagnaro et al., 2012).

The present study is the first to demonstrate that Kefir treatment for 60 days ameliorated the impaired baroreflex sensitivity observed in SHR. When the procedure was repeated under the blockade action of methylatropine the reflex bradycardia and baroreflex gain was abolished in the three groups of animals, indicating that the impaired baroreflex during increases in BP was solely due to the vagal component. On the other hand, when the BP was lowered under the action of the blocker atenolol, the reflex tachycardia was markedly reduced, but this reduction was to a much lesser extent in the Wistar group. Therefore, our data demonstrate that reflex tachycardia is a consequence of both the activation of the cardiac sympathetic component and the simultaneous withdrawn of the parasympathetic activity, which is in agreement with others (Moyses et al., 1994; Campagnaro et al., 2012), and favors the notion that physiological interventions always elicits reciprocal changes in sympathetic and parasympathetic nerve activities. Thus, the tachycardia in response to falls in BP observed could, at least in part, be due to a concurrent vagal activity withdrawal in the Wistar group and possibly in the SHR because the vagal tone was impaired and consequently less effective. In agreement with the review of Head (1994), we observed that the diminished sensitivity of the baroreflex in SHR (characterized by hypertension and cardiac hypertrophy) is mainly due to reduced capacity of the cardiac vagal component rather than a change of the sympathetic (our data: −35 and −23%, respectively). Our data show that kefir treatment of SHR was able to attenuate the impaired baroreflex sensitivity by restoring part of the cardiac autonomic activity. A possible mechanism for the beneficial effect of kefir on the vagal-mediated baroreflex sensitivity in this model of hypertension could be through the reduction of the cardiac hypertrophy as observed by using different probiotics (Lin et al., 2012; Gan et al., 2014; Gómez-Guzmán et al., 2015). Therefore, in addition to other non-pharmacological therapies, such as physical exercise (Ceroni et al., 2009), the chronic administration of the nutritional probiotic kefir appears to have beneficial actions targeting the restoration of the balance between the vagal and sympathetic activities and improving the baroreflex function in hypertensive subjects.

The influence of the autonomic nervous system on the BP and PI is frequency-dependent and the power spectral analysis of beat-to-beat BP recordings has been increasingly used as a valuable non-invasive tool to evaluate the autonomic nervous system modulation on HR and BP in both experimental animals (Murphy et al., 1991; Ceroni et al., 2009; Silva et al., 2009; Hayward et al., 2012; Barbosa Neto et al., 2013) and humans (Silva et al., 2009). We used, in a separated set of experiments, spectral analysis to additionally assess the relative contribution of the sympathetic and vagal tones and their contribution in the reflex activities on conscious SHR chronically treated with kefir when compared with non-treated SHR and normotensive Wistar rats.

In the present study, we observed that kefir significantly reduced the increased cardiac sympathetic tone, which was evaluated through pharmacological blockade and that it reduced the HR variability (or the LF spectra power of PI) in the SHR. It has been demonstrated that LF spectral power of HR is modulated by the autonomic nervous system (Parati et al., 1995; Stauss, 2007; Silva et al., 2009; Chapleau and Sabharwal, 2011). Therefore, a plausible mechanism by which this probiotic partly restores the normal cardiac sympathetic tone and the PI variability may include a direct or indirect inhibition of the sympathetic nerve transmission at its origin in the central nervous system or at the innervation site in the heart. This speculation is corroborated by the finding that the ganglionic blockade (Diedrich et al., 2002) and cardiac muscarinic blockade reduce HR variability (Médigue et al., 2001). Specifically, an important mechanism of action of kefir at the autonomic nervous system could include a decrease in the production of reactive oxygen species and increase of nitric oxide availability, linking the present results to the hypotensive effects of kefir in this genetic model of hypertension, recently reported by Friques et al. (2015).

The PI-LF and BP-LF components, which have been considered markers of cardiac and vascular sympathetic neural activity (Parati et al., 1995; Task Force, 1996), were significantly augmented in SHR when compared with Wistar rats. This finding is consistent with other results (Ceroni et al., 2009; Silva et al., 2009; Hayward et al., 2012; Barbosa Neto et al., 2013). However, the use of LF as an estimative of pure sympathetic activity to the heart has been challenged (Reyes del Paso et al., 2013) and considered a reflection of sympathetic and parasympathetic nerve activities. Increased BP variability is closely correlated to end-organ damage in normotensive and hypertensive animals, and the pharmacologic reduction of BP variability attenuates hypertension outcomes to target-organs (Su and Miao, 2001, 2005). Additionally, our data showed that kefir administered for 60 days to SHR partially corrected the abnormal LF component, suggesting that it could become a non-pharmacological alternative for prevention/treatment of organ-damages associated with increased BP and PI variabilities.

The PI-HF component, which has often been considered a marker of cardiac vagal activity (Dantas et al., 2010), was similar in the Wistar and SHR, consistent with other findings (Ceroni et al., 2009; Barbosa Neto et al., 2013). However, this component does not seem to be solely attributed to cardiac vagal activity, it is also influenced by respiration (Chapleau and Sabharwal, 2011; Reyes del Paso et al., 2013). Additionally, no significant differences were observed among the three groups when we used the LF-to-HF ratio to estimate the sympathovagal balance (Kuwahara et al., 1994; Parati et al., 1995; Task Force, 1996). Kefir treatment did not altered the above parameters. However, the use of the LF/HF ratio as a measure of sympathovagal balance can be misleading (Chapleau and Sabharwal, 2011). The very low frequency (VLF) component was not analyzed in the present study because its physiological correlates are still not clear.

The ratio between the absolute powers of PI-LF and BP-LF, the LF α-index, which has shown to express the spontaneous baroreflex sensitivity (Silva et al., 2009; Dantas et al., 2010, 2012), was diminished in the SHR, which was consistent with other studies (Silva et al., 2009) and the treatment with kefir restored the normal values. As described by Fazan et al. (2005), baroreflex modulation of HR contributes to LF, but not HF variability and is mediated by both sympathetic and parasympathetic drives. Therefore, increased BP variability, which is closely correlated with end-organ damage in hypertensive conditions (Su and Miao, 2001, 2005), can be ameliorated with pharmacological treatment (Su and Miao, 2001; Dias da Silva et al., 2002) and with non-pharmacological interventions, such as with physical exercise (Ceroni et al., 2009) and treatment with flavonoids (Monteiro et al., 2012). Additionally, we demonstrated for the first time that increased BP variability could also account (alternatively or additionally) for beneficial effects of the probiotic kefir, reducing BP variability attenuates end-organ damage. However, we may take into account that, different from the classical pharmacologic approach, the use of variabilities give us only an indirect estimation of the cardiac sympathetic and vagal tone.

The effects of hypertension in target organs could be associated with impaired autonomic function due to increased cerebrovascular oxidative stress and inflammation (Toth et al., 2013). Although our data do not allow us to state precise mechanisms, we speculate that the imbalance between pro- and anti-inflammatory cytokines and between pro- and anti-oxidant molecules could also occur in central areas of neural control of the cardiovascular system, as based on an extrapolation of data from others showing that the hypothalamic paraventricular nucleus (PVN) contributes to sympathoexcitation, hypertension and cardiac hypertrophy in the SHR (Jia et al., 2014). Because kefir is a probiotic beverage with anti-inflammatory (Carasi et al., 2015) and anti-oxidant (Friques et al., 2015) properties, we suggest that the findings of the present study may possible be due to a decreased production of cytokines and reactive oxygen species in the hypothalamic PVN which attenuates hypertension and end-organ damage by up-regulating anti-inflammatory and anti-oxidant molecules, restoring the normal balance (Tan et al., 2015).

In conclusion, the contribution of the cardiac sympathetic and parasympathetic tones and their relative contribution to the baroreflex activities were assessed through classical pharmacologic approaches and spectral analysis. The novelty of this study is that daily administration of kefir for 60 days partly corrects the alterations in cardiac function (including autonomic tone, baroreflex sensitivity) and PI and BP variability in SHR. Therefore, increased BP variability, which is closely correlated with end-organ damage in hypertensive conditions, now could be ameliorate with the beneficial effects of the probiotic kefir, in addition to pharmacological and non-pharmacological therapies.

## Limitations and future directions

A limitation of the present study is that measurement of changes in HR and BP in the protocols we have designed are not sufficient to identify the mechanism of action by which kefir attenuates the abnormalities in the autonomic control of the heart and vessels. Therefore, the present data provide new insights in the prevention/treatment of abnormal vagal and sympathetic control of heart and vessels and opens new avenues for searching the mechanisms involved in the beneficial effects of kefir of the heart and vessels.

## Author contributions

Preparation and administration of kefir: LD and AF; Microbiological analysis of kefir: TA; Hemodynamic measurements: BK and ML; Acquisition and analysis of data from cardiac tones and baroreceptor reflex sensitivity: LD, BK, and AG; Spectral analysis: ED and RD; Critical revision of the manuscript: SM and TP; Conception, study's design, supervision and co-supervision of the study: EV and BC. All authors read and approved the final version of the manuscript.

### Conflict of interest statement

The authors declare that the research was conducted in the absence of any commercial or financial relationships that could be construed as a potential conflict of interest.
